# Influence of Post-Curing in Nitrogen-Saturated Condition on the Degree of Conversion and Color Stability of 3D-Printed Resin Crowns

**DOI:** 10.3390/dj12030068

**Published:** 2024-03-06

**Authors:** Bohyun Lim, Dohyun Kim, Je Seon Song, Sunil Kim, Hoon Kim, Yooseok Shin

**Affiliations:** 1 Department of Conservative Dentistry, College of Dentistry, Yonsei University, Seoul 03722, Republic of Korea; hansie042@yuhs.ac (B.L.); dohyun0kim@yuhs.ac (D.K.); seone1@yuhs.ac (S.K.); 2 Department of Pediatric Dentistry, College of Dentistry, Yonsei University, Seoul 03722, Republic of Korea; songjs@yuhs.ac; 3 Research Institute of Agriculture and Life Sciences, College of Agriculture & Life Sciences, Seoul National University, Seoul 08826, Republic of Korea; c12o2cl4@snu.ac.kr

**Keywords:** degree of conversion, nitrogen, three-dimensional printing, tooth discoloration, post-curing

## Abstract

Post-curing is the process of applying extra light to complete the polymerization process of 3D printing. The mechanical properties of light-cured three-dimensional (3D) printed resin can be improved by decreasing the oxygen concentrations during post-curing, and nitrogen-saturated post-curing has been applied for this purpose. This study aimed to evaluate and compare the color stability of 3D-printed resin crowns that were post-cured in both normal air and nitrogen-saturated conditions. Crowns were fabricated with a 3D printer and post-cured in normal air (control group; air) or nitrogen-saturated conditions (experimental group; nitrogen). The specimens in each group were subdivided into four subgroups, each exposed to different discoloration agents: distilled water, coffee, wine, and curry. Post-immersion color changes were measured using a digital spectrophotometer and analyzed using repeated-measures ANOVA. Fourier transform infrared (FT-IR) spectroscopy evaluated the degree of conversion of resin over immersion times for both post-curing conditions. Upon comparing the effects of post-curing conditions, a significant difference between the control and experimental groups in terms of immersion time in the wine and curry subgroups was found. FT-IR analysis showed a significant difference in the degree of conversion between the air and nitrogen groups from 10 to 300 s. These findings suggest that nitrogen-saturated post-curing can potentially enhance the conversion rate of 3D-printed resin crowns, thereby improving their color stability.

## 1. Introduction

Various three-dimensional (3D) printers have been introduced to fabricate the prostheses that are used in dental practice. Their applications have been increasing in the fields of clinical dentistry due to improvements in their precision and convenience [1]. Most 3D printers use resin compounds to form specific configurations that meet the purpose of restoration [2]. Although their ability to be shaped into proper configurations and their integrity against functional and occlusal force are suitable for clinical use in dental fields, resin compounds are susceptible to discoloration due to their composition and chemical structures [3,4,5,6].

Few studies have demonstrated the limitations of 3D-printed resin restoration in color stability. One study reported changes in the color of 3D-printed resin according to time [7]. In the study, increasing changes in color value (ΔE) in 3D-printed resin were shown according to the time that the specimens were exposed to discoloration agents. Another study also concluded that 3D-printed restorations were more susceptible to discoloration compared to milled restorations and conventional temporization materials [8]. Although these studies demonstrated that 3D-printed resins show color changes, they used disk-shaped specimens which do not entirely reflect the clinical use of 3D-printed resins.

Knowing that 3D-printed resin prostheses are susceptible to discoloration, various methods have been attempted to reduce the discoloration [9,10]. Light-curing varnish was reported to have superior effect over conventional manual polishing technique [11]. Light-cured varnish increases surface smoothness as well as color stability since the resin coating decreases the surface porosity of the interim restoration, infiltrating the restoration’s surface to fill in the micropores and microdefects [9]. However, varnishes are thought to have limitations such as increased total thickness of restoration and probable wear of surface coatings on clinical application.

Another approach is a different method of post-curing. Since the resin compounds acquire their stability and durability by the polymerization reaction, 3D-printed prosthesis needs the process of additional light-curing to ensure the monomers are polymerized to their maximum extent after the initial configuration formation. In this process, the monomers on the surface are influenced by the air that it is in contact with, which forms an oxygen inhibition layer (OIL). OIL is a sticky, resin-rich uncured layer, which is formed due to the high reactivity of oxygen radicals compared to the lower reactivity of monomer radicals [12,13,14]. Thus, the composition of OIL is like that of uncured resin, and the thickness of this layer is known to influence the surface characteristics of resin materials [15]. Recently developed post-curing machines decrease the concentration of oxygen in the air by filling the curing chamber with nitrogen, and it has been reported that post-curing in a nitrogen-saturated condition can reduce the contact of oxygen radicals with the surface of 3D-printed resins and therefore improve their mechanical properties [14,16,17,18]. Also, many studies have demonstrated improvements in the surface characteristics of 3D-printed resins that are post-cured in nitrogen-saturated conditions [19,20,21]. However, no studies regarding the optical properties of 3D-printed resins post-cured under nitrogen-saturated conditions, such as color and translucency, have been reported so far.

Therefore, the purpose of this study was to evaluate the color stability of 3D-printed resin crowns post-cured in nitrogen-saturated conditions and to compare them with crowns that were post-cured in normal air. The null hypothesis was that there would be no difference in the color stability of 3D-printed resin crowns between the two different post-curing conditions: the nitrogen-saturated condition and normal air.

## 2. Materials and Methods

### 2.1. Crown Specimen Preparation

**Figure 1:**
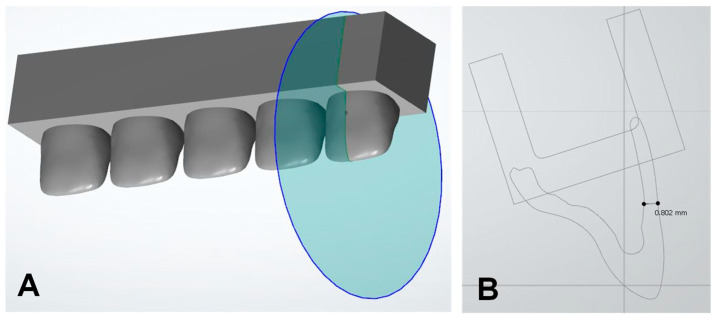
(**A**) CAD design showing five anterior crown configurations combined with specialized supporting structure. (**B**) Cross-section at the mid-facial area of the crown.

Crown specimens were designed using computer-aided design (CAD) software v3.11.3.4 (3Shape Dental System; 3Shape, Copenhagen, Denmark) to make a typical anterior crown for maxillary incisors. The preferred typical incisor crown configuration consists of at least 2 mm of thickness on the incisor edge and 0.7 mm of thickness on the facial surface [22]. Therefore, the crown in this study was constructed with 2.5 mm of thickness on the incisal edge and 0.8 mm of thickness on the facial surface. After design, the crowns were integrated with a specially designed structure on the bottom to ensure that the tip of the color-measuring instrument would fit (Figure 1). This was made to keep the measuring instrument in the same place in the color measurement process. Five crowns were integrated into a specially designed base structure in a row to form a printable form. Two pieces of integrated printable form were fabricated per subgroup to make 10 crown specimens per subgroup.

After the CAD process, the 3D design of the specimen was exported in an STL file, and the specimen was fabricated with a 3D printer (NBEE; Uniz, San Diego, CA, USA) using A2-shaded 3D-printing resin (TC80; Graphy Inc., Seoul, Republic of Korea, FDA number K202846). TC80 consists of dimethacrylic oligomer-blended resin based on urethane acrylate, and this material is cured in the 3D printer using digital light processing (DLP) as the mode of curing. TC80 is a suitable 3D-printing resin used for printing crown and bridge prostheses and it was approved by the FDA. In the initial curing process, resin was cured in its designed configuration layer by layer by the NBEE printer. Then, the specimen was rinsed with 99% isopropyl alcohol (Duksan, Seoul, Republic of Korea) in an ultrasonic bath (UCS-20; Jeiotech, Daejeon, Republic of Korea) for 1 min, according to its manufacturer’s instructions, to remove the excessive resin remnants on the surface of the specimens. The specimens were dried with compressed air and the specimens were divided into two groups depending on the curing process.

Specimens in the air group (control group) were cured in normal air conditions using a post-curing instrument (Tera Harz Cure (THC); Graphy, Seoul, Republic of Korea) for 15 min. The specimens in the nitrogen group were cured in a nitrogen-saturated condition using the same THC curing machine, but with the nitrogen saturation mode enabled, for 15 min. Nitrogen saturation mode is enabled by nitrogen filter machine that is attached to THC. Air with a pressure above 6 atm is supplied to the nitrogen filter machine and a high concentration of nitrogen flows into THC to fill the atmosphere with nitrogen and without oxygen. All specimens were kept in a dark room without any other surface treatment before immersing them in the discoloration agent. A total of 80 specimens were fabricated, and each group consisted of 40 specimens each.

### 2.2. Discoloration Procedures

**Figure 2:**
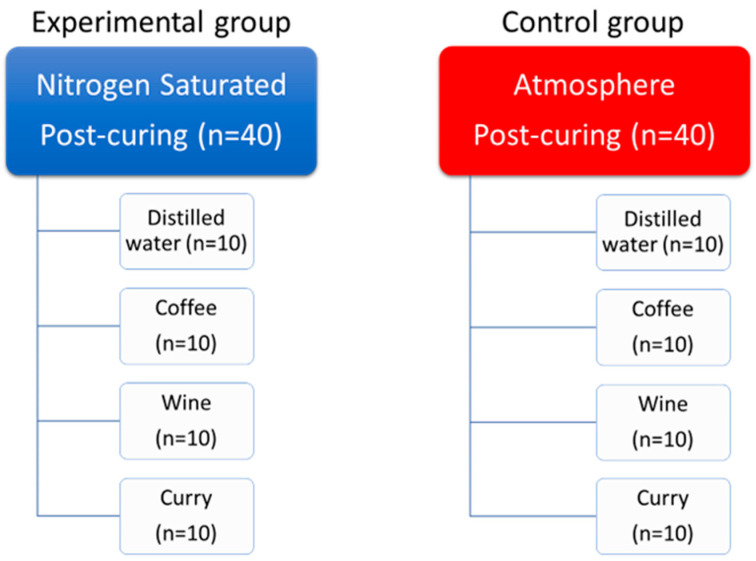
Composition of experimental (nitrogen) and control (air) groups along with the subgroups.

The discoloration agents were selected based on previous studies that studied the color stability of prostheses made using computer-aided design and computer-aided manufacturing (CAD/CAM), and the crown specimens of each group were divided into four subgroups according to the discoloration agents that they were added to, as follows: distilled water, coffee, wine, and curry (Figure 2) [3,4,5,7,8,9]. Coffee was made using Nescafe Crema Americano (Nestle; Vevey, Switzerland) according to the manufacturer’s instructions (3 g of pre-ground powder mixed with 300 mL of distilled water). Wine was Roche Mazet, Cabernet Sauvignon (Groupe CASTEL, Bordeaux, France). Curry was made with Ottogi Curry (Ottogi, Anyang, Republic of Korea) according to the manufacturer’s instructions (25 g of powder mixed with 100 mL of distilled water), and this subgroup was the only subgroup with a hydrophobic main ingredient [23]. The specimens were immersed completely in the discoloration agents in tightly sealed containers. The containers were stored in a 37 °C constant-temperature water bath (HG-10WB; Hangil, Seoul, Republic of Korea) for 4 weeks. They were removed from the discoloration agents for color measurement in each measuring period.

### 2.3. Color Measurement

**Figure 3:**
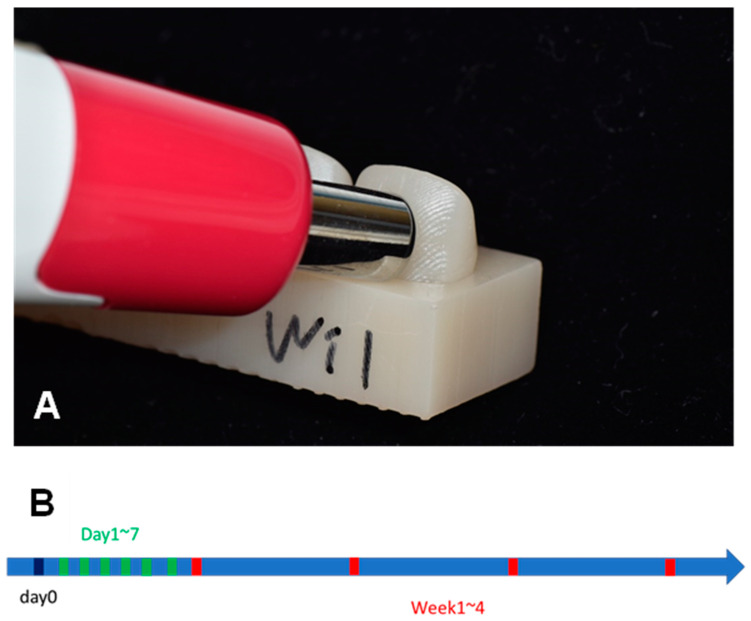
(**A**) Color measurement using a spectrophotometer (Easyshade V). (**B**) Time points when color measurements were performed. Baseline (day 0), every day from 1 to 7 days after immersion, and every week from 1 to 4 weeks after immersion.

The baseline color of each specimen was measured before immersing the specimens in a discoloration agent (Figure 3A). Then, the color of each specimen was measured intermittently every 24 h for a week, followed by once every week for a month (Figure 3B). For each measurement, the specimens were removed from the discoloration agents and thoroughly washed with distilled water. Then, they were dried with compressed air, which made them ready for color measurement.

**Figure 4:**
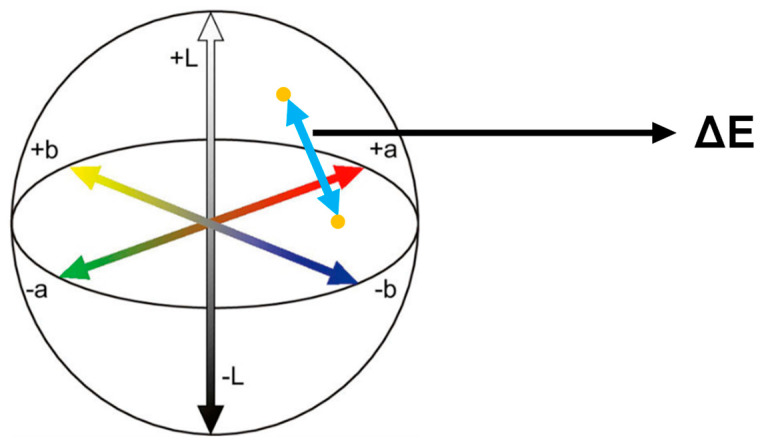
CIELAB color space. The distance between two different colors refers to ΔE.

The color was measured with a portable digital spectrophotometer (EasyShade V; VITA Zahnfabrik, Bad Säckingen, Germany) to acquire the L, a, and b values in the CIELAB color space. L, a, and b values were recorded and ΔE was calculated according to the equation below (Figure 4). (ΔE stands for the distance between the two points on the CIELAB color space).

$$\Delta E=\sqrt{({L_{t}-L_{b})}^{2}+({a_{t}-a_{b})}^{2}+({b_{t}-b_{b})}^{2}}$$

$X_{t}$ = Value of color-orientating factor (*L, ɑ, b*) at the time of measurement.

$X_{b}$ = Value of color-orientating factor (*L, ɑ, b*) at the baseline (day 0).

If the ΔE value was higher than 1.80, it was regarded as clinically perceptible, and if ΔE did not exceed 3.46, it was assumed to be clinically acceptable [24].

### 2.4. Fourier Transform Infrared Spectroscopy Analysis on the Specimen Surface

Fourier transform infrared spectroscopy (FT-IR) analysis was conducted using a Nicolet summit FT-IR spectrometer (Thermo Fisher Scientific, Waltham, MA, USA) with a standard procedure to inspect the degree of conversion of resins according to its curing time and the difference between two post-curing conditions. Using TC80 resin with A2 shade, cylinder-shaped specimens with dimensions of 15 mm of radius and 3 mm of thickness were designed and 3D-printed with a UNIZ 4K Slash2 (UNIZ, San Diego, CA, USA). After fabrication, they were washed with 99% isopropyl alcohol in an ultrasonic bath for 30 s. Specimens in the air group were post-cured in THC from 10 to 900 s without nitrogen saturation mode, and specimens in nitrogen group were post-cured in the same THC but in nitrogen saturation mode. The conversion rates of specimens were recorded at 10, 30, 60, 300, 600, and 900 s after the operation of the post-curing unit. Peaks at 810 cm^−1^ and 1730 cm^−1^ were investigated. The peak at 810 cm^−1^ represents the C=C twisting of acrylate of the uncured resin monomers, and the peak at 1730 cm^−1^ represents the C=O stretching peak, which was used as a reference point to calibrate the peak at 810 cm^−1^ [25,26].

During the measurement, the total quantity of light exposure was also recorded with a UV energy meter (LS128; Linshang, Shenzhen, China). A detector was placed in the middle of the post-curing chamber and the light quantity was measured to match the degree of conversion to the quantity of light.

### 2.5. Statistical Analysis

Statistical analysis was performed with IBM SPSS v26.0 software (IBM, Armonk, NY, USA) at a significance level of 0.05. Repeated-measures one-way analysis of variance (RM-ANOVA) was performed to compare the effect of nitrogen-saturated post-curing on color stability over time. Tukey’s test was performed to evaluate the periods and time duration of significant color changes in the specimens post hoc.

## 3. Results

**Figure 5:**
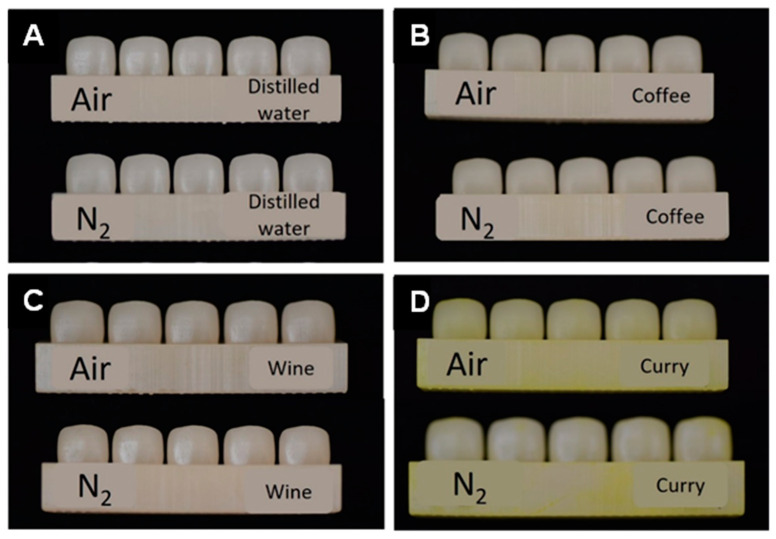
Specimens after a month of immersion in different discoloration agents. (**A**) Distilled water. (**B**) Coffee. (**C**) Wine. (**D**) Curry.

**Figure 6:**
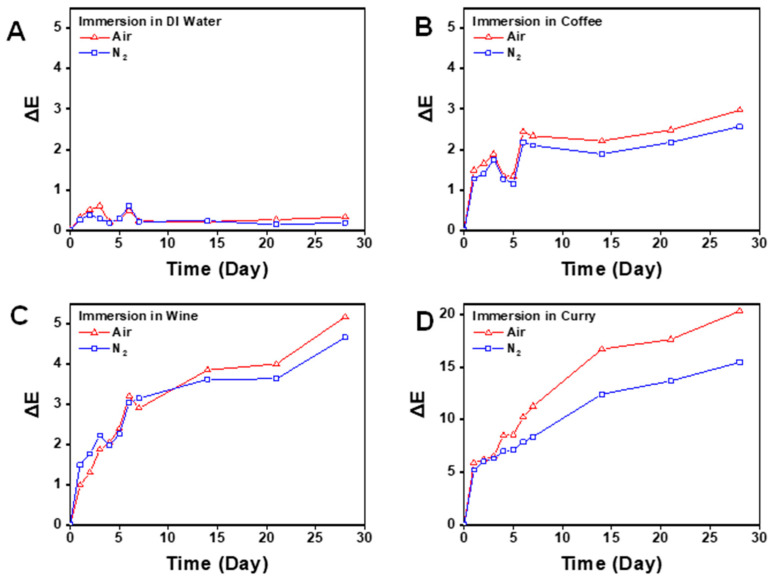
Color change (ΔE) of specimens immersed in different discoloration agents in the control (air) and experimental (nitrogen) groups. (**A**) Distilled water. (**B**) Coffee. (**C**) Wine. (**D**) Curry.

Clinical photos were taken of all specimens after a month of discoloration (Figure 5). Baseline shade showed no significant difference in all experimental groups and control groups regardless of subgroups (*p* < 0.05). All groups and subgroups showed significant differences in color according to the immersion time regardless of curing condition or discoloration agent *(p* < 0.001) (Figure 6). However, when comparing the effect of post-curing methods, the nitrogen group and air group showed no significant differences throughout the experiment in the distilled water and coffee subgroups (*p* > 0.05). The nitrogen group and air group both showed significant differences throughout the immersion time in the wine and curry subgroups (*p* < 0.05). The average value of color change (ΔE) in each specimen at day 3, day 7, and week 4 is described in Table 1.

Also, in the coffee and wine subgroup, the ΔE value of the nitrogen group and air group showed a significant difference in the fourth week only (*p* < 0.05). In the curry subgroup, a significant difference was shown between the nitrogen group and air group at day 5, which continued until the end of the experiment (*p* < 0.05).

The average ΔE value of the nitrogen and air group according to time is shown in Figure 6. Regarding the clinical perceptible standard (ΔE > 1.8), the distilled water subgroup never showed an ΔE value of over 1.8 regardless of the post-curing method. In the coffee subgroup, the air group showed perceptible clinical change right after day 2, while the nitrogen group showed perceptible change after day 5. In the wine subgroup, both the nitrogen group and air group showed a perceptible change from day 3. In the curry subgroup, both groups showed a perceptible change from day 2 and the effect was more distinctive than the other two staining agents.

Regarding the clinically unacceptable standard (ΔE > 3.46), distilled water and coffee subgroups never showed a value that exceeded the acceptable standard in the period of 4 weeks. In wine subgroup, both the nitrogen group and the air group showed an unacceptable change from week 2. In the curry subgroup, both the nitrogen group and the air group showed an unacceptable change from day 1.

**Figure 7:**
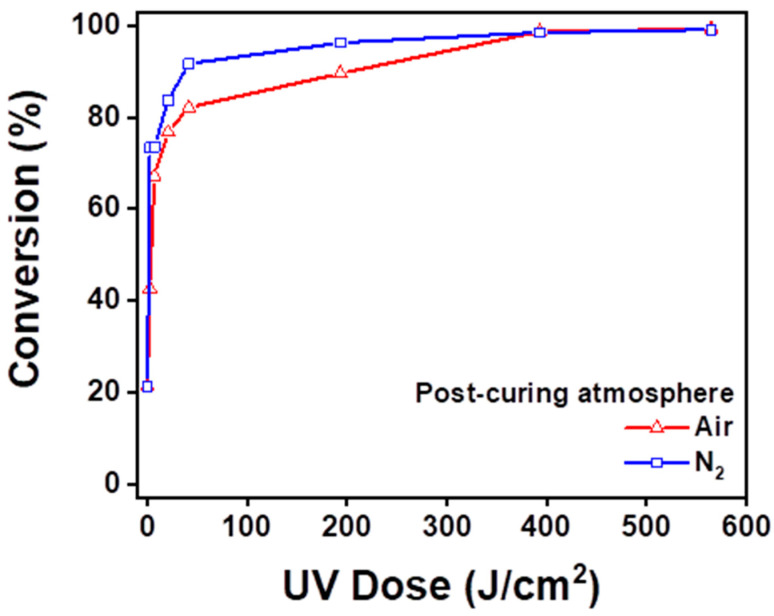
Degree of conversion of resin specimens in each group according to the UV dose.

The degree of conversion was calculated by the volume decrease at the 810 cm^−1^ peak. Figure 7 shows the degree of conversion ratio at 10, 30, 60, 300, 600, and 900 s. The post-curing unit provided the quantity of light during post-curing according to Table 2. The nitrogen group and air group showed significant differences from 10 to 300 s, although the degree of conversion from the air group caught up with the nitrogen group by 600 and 900 s of post-curing time.

## 4. Discussion

All groups and subgroups showed statistically significant differences in color according to the immersion time regardless of curing condition or discoloration agent, which means there was a change in the restoration color according to the time regardless of post-curing methods. This result is in accordance with that of Shin et al., who reported that 3D-printed resins are more susceptible to discoloration [7]. The clinically acceptable limit of color difference (∆E_ab_) is 3.46 [24]. This standard is used as a widely accepted standard in shade measurements of dental prosthesis, as it is cited by various authors since its publication in 2010 and has therefore since been selected as the standard value for evaluation [27,28,29,30,31]. Color differences above the acceptable limit were observed when 3D-printed resins were immersed in wine for 2 weeks or immersed in curry for 1 day.

The reason for this susceptibility to discoloration is thought to be due to the microstructure formed when the resin is cured; 3D-printed prostheses are constructed using additive methods, which inevitably form micro-spaces in between the layers of polymerization [2]. Also, full polymerization is not achieved during the printing session and is delayed until the post-curing session [17]. This delay can lead to a wider space between the layers where discoloration molecules can infiltrate to cause the discoloration of the prothesis.

Another explanation could be water absorption. Water content in the prosthesis can determine the light absorption and reflection pattern of the prosthesis [32]. The shade is perceived by the reflection of lights that is projected onto the prosthesis, and the water absorbed into the resin can affect the difference in L, a, b values. However, this effect would have not been as significant as the spaces between the layers, since 3D-printed resins are less prone to water absorption than conventional interim restoration resin [33]. Also, the distilled water subgroup did not show significant discoloration over time in this study, which implies water absorption alone would not have induced shade change.

The results showed that there was a statistically significant difference in discoloration between the nitrogen-saturated post-curing and normal post-curing methods according to time in the wine and curry subgroups only. Therefore, our null hypothesis is partially rejected. For some discoloration agents like wine and curry, nitrogen-saturated post-curing increased color stability.

Still, at week 4, the distilled water and coffee subgroups also showed a larger ΔE value in the air group, which implies that there might have been a significant difference if the experiment had carried on. This difference in color stability is thought to be due to a greater degree of conversion in the early post-curing process in the nitrogen-saturated condition [16]. As our FT-IR spectroscopy results showed, a significant difference in the degree of conversion was observed during the first 300 s of post-curing. Nitrogen might prevent the formation of an oxygen inhibition layer that inhibits the complete polymerization of monomers on the surface [14]. Although specimens in both the nitrogen group and air group reached a similar degree of conversion after the full five minutes of post-curing, monomers prior to polymerization have a tendency to diffuse from their condensed form, and this delay between initial curing and post-curing can cause more spaces between the monomers, thus making more space for discoloration agents to adhere to the molecular structure.

Several studies have reported improved physical properties of 3D-printed resin prostheses that are cured in nitrogen-saturated conditions. Reymus reported a significantly improved degree of conversion in 3D-printed resin cured in nitrogen-saturated conditions compared to atmospheric conditions, which matches our current FT-IR spectroscopy results [20]. Also, a recent study demonstrated that post-curing in a nitrogen-saturated condition improved Vickers hardness number and degree of double bond conversion [18]. Although surface hardness is not directly related to color stability, it is related to the polymerization rate and degree of conversion on the surface. A harder surface implies a higher degree of polymerization, and this higher polymerization rate could be the reason for color stability, since unpolymerized resin monomers are more susceptible to discoloration. In addition, an observation through SEM revealed that the resin surface post-cured in the nitrogen-saturated condition was smoother than the surface of the resin post-cured in normal air, and this smoother surface might have prevented discoloration agents from easily attaching to the surface [19].

Although most studies of nitrogen-saturated post-curing modes suggest improved physical properties on surfaces, it is suggested that these advantageous effects are only restricted to the surface. If the post-cured surface was removed by a polishing procedure, there was no significant difference in microhardness or degree of conversion rate [21]. This might be obvious, since the nitrogen-saturated atmosphere is only in contact with the surface of the prosthesis. Therefore, if polishing is to be arranged before the delivery of a prosthesis, post-curing in nitrogen conditions might be unnecessary in terms of color stability. Still, the physical properties above are not directly connected to color stability, and further investigation on the color stability itself is needed.

Curry showed the highest discoloration effect compared to other discoloration agents. Also, in our post hoc test, wine exhibited a significant difference only after 4 weeks of immersion time, while curry showed a significant difference after 5 days, which was much faster than the other discoloration agents. The main component that caused discoloration in the curry solution was curcumin. Curcumin is a hydrophobic organic molecule that exhibits keto–enol tautomerism [23]. Unlike coffee and wine, which are acidic and hydrophilic, curcumin has hydrophobic characteristics, and this might have caused greater pigment attachment to the surface of the prosthesis, which is also hydrophobic. The reason why the nitrogen group showed less discoloration might have been due to the dense molecular structure formed from the rapid conversion rate in the nitrogen-saturated condition, as stated previously.

The current study investigated the color stability of 3D-printed resin cured in a nitrogen-saturated condition, whereas most previous studies focused on the physical properties and degree of conversion. Post-curing in a nitrogen-saturated condition has been researched only in 3D-printed occlusal splints in previous studies, so this study might play a pioneering role in color stability of 3D-printed prosthesis. Also, the specimens used in this study were fabricated according to their actual clinical use in crown configuration. This configuration mimics the actual clinical situation where these 3D-printed protheses are used and may present more clinically practical data. In addition, the discoloration agents in this study include curry, which is an unusual discoloration agent that is not used in previous studies. Unlike other discoloration agents, curry is hydrophobic, and this simulates clinical conditions where some diets are hydrophobic. Concerning FT-IR analysis, no other study has reported the quantity of light according to the time of curing. This procedure allowed the researcher to keep track of the degree of conversion change in certain periods of curing. These data can provide a better understanding of the polymerization process of resin monomers.

However, this study also presented many limitations. The color measurement instrument might not have made perfect contact with the specimens, since the veneer crown configuration has curves and the instrument tip is flat. This might have led to measuring errors in the study. Also, the specimens were not tested in real clinical situations where saliva acts as a cleansing material, and the impact of brushing teeth was not analyzed in the experiment. In these conditions, the resin prosthesis might have experienced less discoloration than in a real clinical situation. Thus, since this study was only limited to in vitro conditions, additional study is needed to assess the efficacy of nitrogen-saturated post-curing in clinical applications.

With the results shown in this research, clinicians can utilize a nitrogen-saturated post-curing technique to improve the color stability of a 3D-printed prosthesis. Color stability of such a prosthesis can significantly affect the satisfaction of the patient who has an interest in aesthetics.

## 5. Conclusions

Within the limitations of this current study, the following were concluded:

1. Our 3D-printed crowns were susceptible to discoloration in various discoloration agents, and the discoloration increased with the time of contact.
2. Although post-curing in a nitrogen-saturated condition improved the color stability of the 3D-printed resin crowns, the change in color may not be acceptable clinically with both wine and curry consumption.
3. On the surface of the 3D-printed crowns, the degree of conversion reached its full extent faster in nitrogen-saturated conditions compared to in normal air curing conditions.

## Figures and Tables

**Table 1:** Average of color change (ΔE) in each specimen at day 3, day 7, and week 4.

Subgroups/Periods	Nitrogen	Air	*p*
**Distilled water**			
Day 3	0.3 ± 0.1	0.6 ± 0.2	0.625
Day 7	0.5 ± 0.5	0.4 ± 0.3	0.780
Week 4	0.4 ± 0.2	0.5 ± 0.2	0.157
**Coffee**			
Day 3	1.8 ± 0.4	2.0 ± 0.5	0.274
Day 7	2.1 ± 0.4	2.4 ± 0.4	0.094
Week 4	2.6 ± 0.3	3.0 ± 0.3	0.004 *
**Wine**			
Day 3	2.2 ± 0.6	1.9 ± 0.4	0.145
Day 7	3.2 ± 0.4	2.9 ± 0.3	0.170
Week 4	4.7 ± 0.6	5.1 ± 0.5	0.043 *
**Curry**			
Day 3	6.3 ± 0.8	6.6 ± 0.9	0.596
Day 7	8.3 ± 0.8	11.3 ± 0.8	0.000 *
Week 4	15.4 ± 1.2	20.3 ± 2.2	0.000 *

* Statistically significant (*p* < 0.05) as determined by Tukey’s comparison test.

**Table 2:** Quantity of light that the specimens were exposed to according to the post-curing time.

Seconds	10	30	60	300	600	900
J/cm^2^	6.9	20.7	41.4	193.1	397.7	564.3

## Data Availability

Data are contained within the article.
